# Pre-Treatment *SEPTIN9* Gene Methylation Ratio Predicts Tumor Response to Total Neoadjuvant Therapy in Patients with Locally Advanced Rectal Cancer

**DOI:** 10.3390/cancers17060965

**Published:** 2025-03-13

**Authors:** Víctor Domínguez-Prieto, Miguel León-Arellano, Rocío Olivera-Salazar, Luz Vega-Clemente, Cristina Caramés, Eva Ruiz-Hispán, Raquel Fuentes-Mateos, Diana Rosero-Rodríguez, Héctor Guadalajara, Mariano García-Arranz, Damián García-Olmo

**Affiliations:** 1 Surgery Department, Fundación Jiménez Díaz University Hospital, 28040 Madrid, Spain; miguel.leon@quironsalud.es (M.L.-A.); h.guadalajara@quironsalud.es (H.G.); damian.garcia@quironsalud.es (D.G.-O.); 2 New Therapies Laboratory, Fundación Jiménez Díaz Health Investigation Institute, Fundación Jiménez Díaz University Hospital, 28040 Madrid, Spain; rocio.olivera@quironsalud.es (R.O.-S.); luz.vega@quironsalud.es (L.V.-C.); mariano.garcia@quironsalud.es (M.G.-A.); 3 Oncology Department, Fundación Jiménez Díaz University Hospital, 28040 Madrid, Spain; ccarames@quironsalud.es (C.C.); eva.ruizh@quironsalud.es (E.R.-H.); raquel.fmateos@quironsalud.es (R.F.-M.); diana.rosero@quironsalud.es (D.R.-R.); 4 Faculty of Medicine, Universidad Autónoma de Madrid, 28029 Madrid, Spain

**Keywords:** rectal cancer, locally advanced rectal cancer, *SEPTIN9* gene methylation ratio, tumor response, total neoadjuvant therapy

## Abstract

**Background and objective:** Multiple markers have been proposed, but there are no reliable pre-treatment markers that predict tumor response to total neoadjuvant therapy in patients with locally advanced rectal cancer. The objective of this study is to evaluate the usefulness of pre-treatment *SEPTIN9* gene methylation ratio as a predictor of tumor response to total neoadjuvant therapy and its correlation with tumor size and tumor stage in patients with locally advanced rectal cancer. **Methods:** Patients with locally advanced rectal cancer (T3/4 and/or N+ histologically confirmed rectal cancer) undergoing total neoadjuvant therapy were included. Tumor size and tumor stage were determined by magnetic resonance. *SEPTIN9* gene methylation in plasmatic cfDNA was analyzed by droplet digital PCR at the time of diagnosis. After completing total neoadjuvant therapy, tumor response was assessed by magnetic resonance and proctoscopy. The correlation between pre-treatment *SEPTIN9* gene methylation ratio, tumor size, tumor stage and tumor response was analyzed. **Results:** 39 patients with locally advanced rectal cancer were included. Pre-treatment SEPTIN9 gene methylation ratio (*p* = 0.033) and tumor size (*p* = 0.026), but not tumor stage, significantly correlated with tumor response to total neoadjuvant therapy. Pre-treatment SEPTIN9 gene methylation ratio also correlated with N stage (*p* = 0.040) and tumor size (*p* = 0.001), but not with T stage (*p* = 0.846). **Conclusions:** Pre-treatment SEPTIN9 gene methylation ratio correlates with tumor size and N stage and can predict tumor response to total neoadjuvant therapy in patients with locally advanced rectal cancer.

## Simple Summary

There are no reliable pre-treatment markers that predict tumor response to total neoadjuvant therapy in patients with locally advanced rectal cancer. The aim of this study is to evaluate the usefulness of pre-treatment *SEPTIN9* gene methylation ratio as a predictor of tumor response to total neoadjuvant therapy and its correlation with tumor size and tumor stage in patients with locally advanced rectal cancer. Pre-treatment *SEPTIN9* gene methylation ratio (*p* = 0.033) and tumor size (*p* = 0.026), but not tumor stage, significantly correlated with tumor response to total neoadjuvant therapy. Pre-treatment *SEPTIN9* gene methylation ratio also correlated with N stage (*p* = 0.040) and tumor size (*p* = 0.001), but not with T stage (*p* = 0.846). Thus, pre-treatment *SEPTIN9* gene methylation ratio correlates with tumor size and N stage and can predict tumor response to total neoadjuvant therapy in patients with locally advanced rectal cancer.

## 1. Introduction

Colorectal cancer (CRC) is the third most common cancer in the world, while rectal cancer (RC) accounts for approximately 732,000 new cases per year, according to data from the *American Society of Clinical Oncology* [1].

Locally advanced rectal cancer (LARC) is defined as T3/4 and/or N+ rectal cancer. The standard treatment of LARC, which includes neoadjuvant chemoradiation followed by radical surgery with total mesorectal excision and adjuvant chemotherapy, achieves good survival rates and excellent local control of the disease, with low rates of local recurrence but with remaining high rates of distant metastasis [2]. Furthermore, surgical resection involves important morbidity that significantly affects quality of life [3].

Consequently, during the last decades, there has been a growing interest in organ preservation approaches to avoid the morbidity derived from surgery in those patients with a complete clinical response (cCR) to neoadjuvant treatment. Total neoadjuvant therapy (TNT) has demonstrated achievements of better systemic control of the disease and increased rates of cCR, which has led to avoiding surgery in those patients with a cCR in a “watch and wait” (WW) follow-up protocol [2].

Tumor response to neoadjuvant chemoradiation is an important prognostic factor in patients with LARC. Multiple markers have been proposed, but no reliable pre-treatment markers that predict tumor response to TNT in patients with LARC have been developed enough to reach regular clinical use, since all of them have important limitations [4].

*SEPTIN9* gene methylation (SEPT9m) is an epigenetic phenomenon that occurs during CRC tumorigenesis and a well-demonstrated biomarker in plasma for screening and early diagnosis of CRC [5]. Moreover, it has also demonstrated its utility as a biomarker of complete surgical resection and tumor recurrence during the follow-up after surgery in patients with CRC [6,7].

To our knowledge, its value as a predictor of tumor response to TNT in patients with LARC has not been explored. Thus, the objective of this study is to evaluate the usefulness of the pre-treatment *SEPTIN9* gene methylation ratio (SEPT9mr) as a predictor of tumor response to TNT and its correlation with tumor size and tumor stage in patients with LARC, since we hypothesize that pre-treatment SEPT9mr should be correlated and could predict tumor response to TNT.

## 2. Materials and Methods

### 2.1. Patients’ Selection

Patients with histologically confirmed LARC (T3/4 and/or N+ RC) included in a TNT protocol with the goal of organ preservation in Fundación Jiménez Díaz University Hospital were enrolled in this study after discussion in a multidisciplinary committee.

### 2.2. LARC Diagnosis

Diagnosis of LARC was made by digital rectal examination (DRE), magnetic resonance imaging (MRI), and proctoscopy with a biopsy positive for adenocarcinoma. Tumor size (largest tumor diameter) and tumor stage at diagnosis were determined by MRI. A thoracic-abdominopelvic CT (Siemens, Munich, Germany) scan confirmed the absence of distant metastases at the time of diagnosis in each case.

### 2.3. Treatment of LARC

After the diagnosis of LARC, patients received one of the following schemes of TNT with the goal of organ preservation:

Chemoradiation with short-or long-course radiotherapy and concomitant sensitizing chemotherapy with capecitabine followed by 6–8 cycles of consolidation systemic chemotherapy with FOLFOX (oxaliplatin, 5-fluorouracil, and folinic acid).

Induction chemotherapy with FOLFOX was followed by chemoradiation with short- or long-course radiotherapy and concomitant sensitizing chemotherapy with capecitabine.

### 2.4. Evaluation of Tumor Response to TNT

After completing TNT, evaluation of tumor response was performed by DRE, proctoscopy, and MRI. Tumor response was graded following MRI Tumor Regression Grade (mrTRG) as follows [8]:

**mrTRG 1**: Complete response. No evidence of tumor signal intensity or fibrosis only.

**mrTRG 2**: Good response. Dense fibrosis or minimal residual tumor.

**mrTRG 3**: Moderate response. Mixed fibrosis/mucin and intermediate signal representing residual tumor, but fibrosis still predominates.

**mrTRG 4**: Slight response. Small areas of fibrosis or mucin but mostly tumor.

**mrTRG 5**: No response. Same appearance as original tumor or tumor growth.

Patients with a grade 1–2 mrTRG and a negative proctoscopy were supposed to have a cCR, were included in a WW protocol, and closely followed by DRE, CT scan, MRI, and proctoscopy. Patients with a grade 3–5 mrTRG and/or a positive proctoscopy, who did not reach a cCR, were indicated for surgery (either abdominoperineal amputation, anterior low resection of the rectum or transanal local resection).

### 2.5. SEPT9m Analysis

For each patient, pre-treatment SEPT9m in plasmatic cell-free DNA (cfDNA) was analyzed by droplet digital polymerase chain reaction (ddPCR).

At the time of LARC diagnosis, before starting TNT, 20 mL of whole peripheral blood was collected from each patient in 2 EDTA tubes. Plasma was isolated within a maximum of 2 h through two centrifugation steps: first at 1800 G, 4 °C, for 10 min to eliminate cell debris, followed by a second centrifugation at 3000 G, 4 °C, for 10 min. Isolated plasma was then stored frozen at −80 °C for further analysis.

cfDNA isolation from plasma was performed using QIAamp Circulating Nucleic Acid Kit (Qiagen, Hilden, Germany). Isolated cfDNA was afterward treated with an EZ DNA Methylation Kit by spin column (Zymo Research, Irvine, CA, USA).

SEPT9m in cfDNA was analyzed by the ddPCR assay QX200 Droplet Digital PCR System (Bio-Rad Laboratories, Hercules, CA, USA). Samples were prepared by mixing 10 μL of ddPCR Supermix for probes No dUTP (Bio-Rad Laboratories, Hercules, CA, USA), 1 μL of FAM and HEX fluorescent probes (specific for mutant/methylated and wild-type gene, respectively), and 1 to 8.8 μL of template DNA in a final reaction volume of 20 μL. Three replicates were analyzed per sample. Distillated water instead of DNA was used for no template control and served as a control for detecting environmental contamination. DNA from HCT-116 cell line, which has hypermethylation of the *SEPTIN9* gene, was used as positive control.

Primers for *SEPTIN9* gene (5′-AGAGAATTTTGTTTGGTTGTTGTTTAAATATATAG-3′ and 5′-AAAAAAAAAATTCCTCCCCTTCC-3′) (Bio-Rad Laboratories, Hercules, CA, USA) and fluorescent probes for the methylated and unmethylated sequences (methylated-FAM 5′-TGTAGAAGGATTTTGCGTTCGG-3′ unmethylated-HEX 5′-TTGTAGAAG/ZEN/GATTTTGTGTGTTTGG-3′) were used once the DNA was converted with bisulfite. A total of 40 cycles of ddPCR were employed at 95 °C for 10 min including 94 °C for 30 s and 52 °C for 1 min for amplification, a final cycle at 98 °C for 10 min, and a holding temperature of 4 °C.

**Figure 1:**
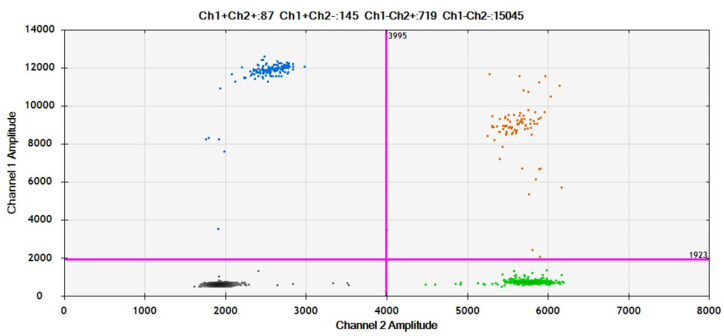
Example of SEPT9m analysis by ddPCR: Blue: drops with methylated SEPTIN9 gene. Orange: drops with methylated + unmethylated SEPTIN9 gene. Green: drops with unmethylated SEPTIN9 gene. Black: no template control.

Droplets were generated by a QX200 droplet generator (Bio-Rad Laboratories, Hercules, CA, USA), and endpoint PCR was performed on a T100 Thermal Cycler (Bio-Rad Laboratories, Hercules, CA, USA). After thermal cycling, the fluorescent signals of droplets were detected in the FAM and HEX channels of a QX200 droplet reader (Bio-Rad Laboratories, Hercules, CA, USA). Data were analyzed by Quanta Soft v.1.7 Software (Bio-Rad Laboratories, Hercules, CA, USA). Results were reported as the number of copies of genetic alteration per μL of reaction (Figure 1).

We previously observed that analyzing the absolute value of analytes from global plasma samples is highly variable to reach an objectively assessable result. Therefore, we considered the individual ratio of methylated to unmethylated *SEPTIN9* gene, which we called “*SEPTIN9* gene methylation ratio” (SEPT9mr), to be more consistent and objective. SEPT9mr was therefore calculated as follows:

(1) $$SEPT9mr=\frac{copies\;with\;methylated\;SEPTIN9\;gene}{copies\;with\;unmethylated\;SEPTIN9\;gene}$$

## 3. Results

A total of 39 patients (61.5% male, 38.5% female) were included from December 2020 to January 2024. The median age was 63.3 (38–80) years.

The median tumor size was 4.3 cm, ranging from 2 to 8.5 cm. Regarding tumor stage, 2 patients (5.1%) had a T2 stage, 31 patients (79.5%) had a T3 stage, and 6 patients (15.4%) had a T4 stage. A total of 4 patients (10.3%) had an N0 stage, 20 patients (51.3%) had an N1 stage, and 15 patients (28.5%) had an N2 stage. The median SEPT9mr at the time of diagnosis was 0.0317. (Table 1).

A total of 23 (59%) patients achieved a cCR and enrolled in a WW protocol. A total of four (10.3%) patients were not supposed to achieve a cCR and underwent surgery, but the histopathological analysis of the surgical specimen revealed the absence of tumor, so they were considered as complete tumor response (cTR) because a complete pathological response (cPR) was demonstrated. Thus, 27 (69.2%) patients in total obtained a cTR. The remaining 12 (30.8%) patients did not achieve a cCR and were indicated either for radical or local resection, which confirmed the absence of cTR *(*Table 2).

Both groups (patients with and without cTR) were comparable in terms of sex and age. A statistically significant lower SEPT9mr was observed in the group of patients who achieved a cTR to TNT, compared with those who did not achieve a cTR (0.023 vs. 0.032, *p* = 0.033), so higher values of SEPT9mr seem to predict a lower probability of cTR. Significant differences in tumor size (4.0 vs. 4.9 cm, *p* = 0.026), but not in tumor stage, between patients with and without cTR to TNT were also observed, so greater tumors seem to correlate with a lower likelihood of cTR (Table 3).

SEPT9mr also shows a statistically significant correlation with the N stage (*p* = 0.040) but not with the T stage (*p* = 0.846) in a Kruskal–Wallis test, and there is a moderate correlation with tumor size (Spearman’s correlation coefficient = 0.54, *p* = 0.001).

**Figure 2:**
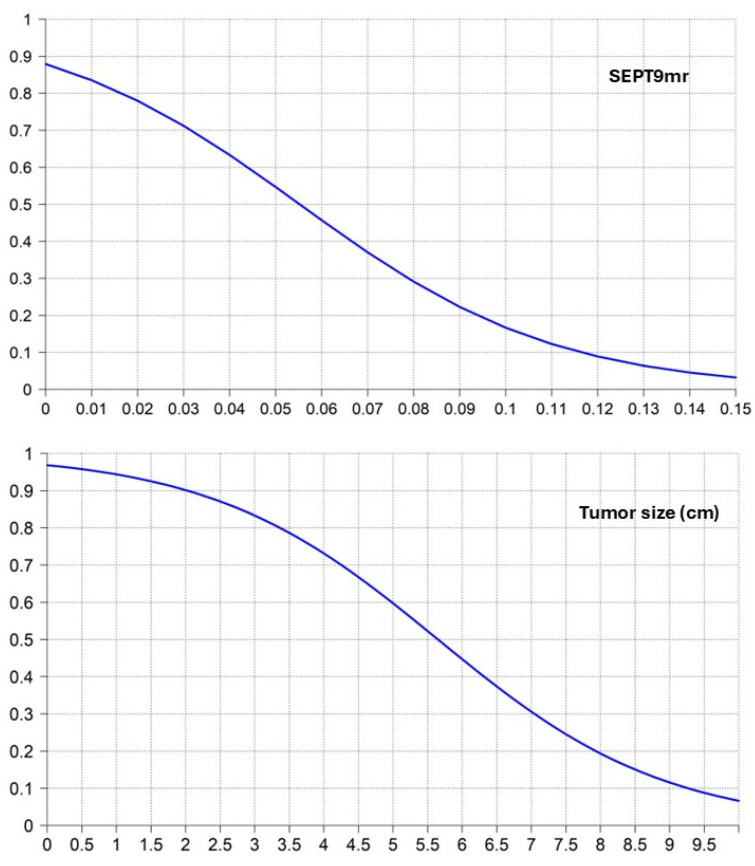
Logistic regression curves of SEPT9mr and tumor size predicting the likelihood of cTR.

The likelihood of cTR depending on SEPT9mr and tumor size was explored in a logistic regression analysis (Figure 2) (Table 4).

## 4. Discussion

Human carcinogenesis not only depends on genetic changes but also on epigenetic alterations like DNA methylation. Thus, aberrant DNA hypermethylation in the promoter region of some genes may lead to inappropriate gene silencing and cancer development [9]. *SEPTIN9* is a gene located in chromosome 17q25 that codifies filamentous proteins involved in microtubule formation, angiogenesis, cell motility, cell proliferation, and cell cycle control, among others [9,10,11].

*SEPTIN9* gene hypermethylation is one of the most studied epigenetic changes, given its implication in CRC carcinogenesis [9,11], and a well-known biomarker for CRC diagnosis and follow-up. High specificity and negative predictive value, and moderate sensitivity have been de-scribed for the early detection of CRC [5]. Given its significant decrease after surgery [12,13], it has demonstrated its utility as a biomarker of complete surgical resection [6], minimal residual disease, and tumor recurrence after surgery in patients with CRC [7,13]. It has also been proposed as a potential biomarker of antitumor therapy effectiveness since SEPT9m levels in plasma decrease by 1.7 times after chemotherapy and 2.3 times after RC resection [14]. Moreover, the plasmatic levels of SEPT9m are quantitatively correlated with tumor burden in patients with stage IV CRC and correlated with prognosis in terms of survival [15].

Tumor response to neoadjuvant chemoradiation is an important prognostic factor in patients with LARC [4], but the prediction of tumor response to TNT is a difficult setting. Multiple markers have been proposed, both pre- and post-neoadjuvant chemoradiation, with varied results: gene expression analysis; patient-derived organoids; single-nucleotide polymorphisms (SNPs); DNA methylation; microRNAs; circulating tumor DNA (ctDNA); carcinoembryonic antigen (CEA); circulating tumor cells; tumor size; T and N stage; tumor distance from the anal verge; circumferential extent; anterior tumor position; intratumoral budding; tumor microenvironment; mucinous histology; poor tumor differentiation; macroscopic ulceration; gut microbiome; lymphovascular invasion, perineural invasion, and absence of necrosis; tumor–stroma ratio; lymphocyte–monocyte ratio; body mass index; hemoglobin levels; PET/CT standardized uptake value (SUV); radiomics; time after chemoradiation; *APC*, *PIK3CA*, *TP53*, *SMAD4*, *KRAS*, *NRAS*, and *BRAF* mutations; pro-apoptotic factor Bax; the anti-apoptotic X-linked inhibitor of apoptosis protein (XIAP); VEGF; hypoxia-inducible protein factor-1 (HIF-1); osteopontin; myoferlin; fatty acid metabolism; and cytokines like IL-1 and IL-8… [4,16,17,18,19,20,21,22,23,24,25,26,27,28,29,30,31,32,33,34,35,36]. But no reliable pre-treatment markers that predict tumor response to TNT in patients with LARC have been developed enough to reach regular clinical application, since all of them have important limitations [4].

One of the most employed biomarkers for diagnosis and follow-up of CRC is CEA, since its elevation is considered a strong marker of recurrence [37]. Nevertheless, CEA is not a specific marker of CRC, so it can be elevated in other digestive neoplasms and inflammatory diseases [37], and 70% of patients with RC have negative CEA levels [38]. Despite the fact that CEA does not seem to be a reliable marker for these settings, low CEA levels (≤5 ng/mL) have been associated with an increased likelihood of cPR to neoadjuvant chemoradiation in patients with LARC in some studies [35].

Therefore, there is an imperative need for the development of new biomarkers that could solve these limitations and predict tumor response to TNT. According to our results, pre-treatment SEPT9mr in plasmatic cfDNA significantly correlates with tumor size, N stage and tumor response to TNT, so higher values of SEPT9mr seem to predict a lower probability of cTR. Therefore, based on the pre-treatment SEPT9mr that we could select, at the time of diagnosis, those patients who will respond to TNT. Tumor size, but not tumor stage, also correlates with tumor response to TNT, so greater tumors seem to correlate with a lower likelihood of cTR.

It is important that there are no statistically significant differences in age between both groups (patients with and without a cTR), since DNA methylation is a physiological phenomenon that also increases with aging [39,40].

One of the main advantages of SEPT9mr compared with other SEPT9m tests is that it brings a quantitative, continuous, non-dichotomic, and relative value of the degree of methylation of *SEPTIN9* gene that can also be monitored during LARC treatment and follow-up.

Nevertheless, these results must be taken with caution due to the relatively small sample size analyzed and validated in forthcoming studies, including larger, independent cohorts.

## 5. Conclusions

Pre-treatment SEPT9mr significantly correlates with tumor size, N stage, and the likelihood of cTR to TNT in patients with LARC. Therefore, SEPT9mr seems to be a valuable pre-treatment biomarker for predicting tumor response to TNT.

## Figures and Tables

**Table 1:** Demographic and tumor characteristics.

**Age (Years)**	63.3 (38–80)
**Sex**	
**Male**	24 (61.5%)
**Female**	15 (38.5%)
**Tumor size (cm)**	4.3 (2–8.5)
**T stage**	
**T1**	0 (0%)
**T2**	2 (5.1%)
**T3**	31 (79.5%)
**T4**	6 (15.4%)
**N stage**	
**N0**	4 (10.3%)
**N1**	20 (51.3%)
**N2**	15 (38.5%)
**SEPT9mr**	0.0317 (0.008–0.125)

**Table 2:** Tumor response to TNT.

**cTR**	**cCR**	23 (59%)	
	**Non-cCR but cPR**	4 (10.3%)	27 (69.2%)
**Non-cTR**	**Non-cCR nor cPR**	12 (30.8%)	12 (30.8%)

**Table 3:** Correlation between age, sex, tumor size, tumor stage, SEPT9mr, and tumor response to TNT.

	cTR		*p*	OR (95% CI)
	No	Yes		
**Age (years)**	62.0 ± 12.2	63.9 ± 10.5	0.624	1.02 (0.95, 1.08)
**Sex**			0.580	
**Male**	6 (50.0%)	18 (66.7%)		
**Female**	6 (50.0%)	9 (33.3%)		
**SEPT9mr**	0.032 (0.023–0.043)	0.023 (0.018–0.029)	0.033	0.97 (0.92, 1.00)
**Tumor size (cm)**	4.9 (4.0–6.7)	4.0 (2.9–4.5)	0.026	0.55 (0.30, 0.88)
**T**			0.748	
**T2**	1 (8.3%)	1 (3.7%)		
**T3**	8 (66.7%)	23 (85.2%)		
**T4**	3 (25.0%)	3 (11.1%)		
**N**			0.368	
**N0**	0 (0.0%)	4 (14.8%)		
**N1**	6 (50.0%)	14 (51.9%)		
**N2**	6 (50.0%)	9 (33.3%)		

**Table 4:** Logistic regression analysis of SEPT9mr and tumor size predicting the likelihood of cTR.

SEPT9mr	Probability of cTR	Tumor Size (cm)	Probability of cTR
0.00	0.879	0.0	0.969
0.01	0.835	0.5	0.958
0.02	0.780	1.0	0.944
0.03	0.712	1.5	0.925
0.04	0.633	2.0	0.902
0.05	0.547	2.5	0.871
0.06	0.457	3.0	0.833
0.07	0.370	3.5	0.787
0.08	0.291	4.0	0.731
0.09	0.223	4.5	0.668
0.10	0.167	5.0	0.597
0.11	0.122	5.5	0.523
0.12	0.089	6.0	0.447
0.13	0.064	6.5	0.374
0.14	0.045	7.0	0.306
0.15	0.032	7.5	0.245
		8.0	0.194
		8.5	0.151
		9.0	0.116
		9.5	0.088
		10.0	0.067

## Data Availability

The raw data supporting the conclusions of this article will be made available by the authors upon request.

## Abbreviations

CRC	Colorectal cancer
RC	Rectal cancer
LARC	Locally advanced rectal cancer
cCR	Complete clinical response
TNT	Total neoadjuvant therapy
WW	Watch and wait
SEPT9m	SEPTIN9 gene methylation
SEPT9mr	SEPTIN9 gene methylation ratio
DRE	Digital rectal examination
MRI	Magnetic resonance imaging
mTRG	MRI Tumor Regression Grade
cfDNA	Cell-free DNA
ddPCR	Droplet digital polymerase chain reaction
cTR	Complete tumor response
cPR	Complete pathological response
CEA	Carcinoembryonic antigen
